# Knee disorders in primary care: design and patient selection of the HONEUR knee cohort

**DOI:** 10.1186/1471-2474-6-45

**Published:** 2005-08-23

**Authors:** Edith M Heintjes, Marjolein Y Berger, Bart W Koes, Sita M Bierma-Zeinstra

**Affiliations:** 1 Department of General Practice, Erasmus University Medical Centre, P.O. Box 1738, 3000 DR Rotterdam, The Netherlands

## Abstract

**Background:**

Knee complaints are a frequent reason for consultation in general practice. These patients constitute a specific population compared to secondary care patients. However, information to base treatment decisions on is generally derived from specialistic settings. Our cohort study is aimed at collecting knowledge about prognosis and prognostic factors of knee complaints presented in a primary care setting. This paper describes the methods used for data collection, and discusses potential selectiveness of patient recruitment.

**Methods:**

This is a descriptive prospective cohort study with one-year follow-up. 40 Dutch GPs recruited consecutive patients with incident knee complaints aged 12 years and above from October 2001 to October 2003. Patients were assessed with questionnaires and standardised physical examinations. Additional measurements of subgroups included MRI for recent knee traumas and device assessed function measurements for non-traumatic patients.

After the inclusion period we retrospectively searched the computerized medical files of participating GPs to obtain a sample to determine possible selective recruitment. We assessed differences in proportions of gender, traumatic onset of injury and age groups between participants and non-participants using Odds Ratios (OR) and 95% confidence intervals.

**Results:**

We recruited 1068 patients. In a sample of 310 patients visiting the GP, we detected some selective recruitment, indicating an underrepresentation of patients aged 12 to 35 years (OR 1.70; 1.15–2.77), especially among men (OR 2.16; 1.12–4.18). The underrepresentation of patients with traumatic onset of injury was not statistically significant.

**Conclusion:**

This cohort is unique in its size, setting, and its range of both age and type of knee complaints. We believe the detected selective recruitment is unlikely to introduce significant bias, as the cohort will be divided into subgroups according to age group or traumatic onset of injury for future analyses. However, the underrepresentation of men in the age group of 12 to 35 years of age warrants caution. Based on the available data, we believe our cohort is an acceptable representation of patients with new knee complaints consulting the GP, and we expect no problems with extrapolation of the results to the general Dutch population.

## Background

Knee complaints rank among the most frequent reasons for consulting primary care physicians. A nationwide study into the incidence and prevalence of diseases and complaints in Dutch General Practices revealed that the incidence of unspecified knee complaints in General Practice is 13.7 per 1000 patients per year, ranking 16^th ^in the list of most frequent reasons for visiting the General Practitioner (GP). Specified knee complaints (knee distortion, acute injury to meniscus or ligaments, chronic internal traumatic knee injuries, knee osteoarthritis, and Osgood Schlatter) account for an incidence 11.3 per 1000 on top of that [1].

Nonetheless, clinical research in this area is usually carried out in hospital settings and only covers serious or persistent injuries, usually meeting stringent inclusion criteria. The applicability of results from this research to patients presenting knee complaints in general practice is therefore limited. Open population studies [2,3] offer a broader view of knee complaints, but often target specific age groups and also include patients that do not seek medical care for their complaints. To our knowledge, publication of studies dealing with patients with knee disorders in general practice is limited to cross-sectional registration studies that report incidence and prevalence of diagnostic codes and their corresponding referral rates to physical therapy or specialist care [1]. This type of study is not informative with respect to disease burden, the (natural) course of complaints, treatments strategies or even diagnosis, because the diagnostic codes are often non-specific. As a result, our understanding of knee complaints in primary care is far from complete. But knowledge about the determinants of the clinical course is essential for making management decisions and to inform patients about their prognosis. Furthermore, decisions about management and referral of knee complaints in primary care are to a large extent based on test results from physical examination. Physical signs and symptoms may also play an important part in predicting the course of knee complaints. Nevertheless, the value of physical examination in general practice has never been evaluated.

To fill in the gaps in the information available to GPs, we performed a prospective, observational cohort study including the whole range of incident knee complaints presented to the GP, by adolescents as well as adults. The primary objectives of our cohort study are as follows:

1. What type of knee complaints are presented to the GP, and what is their severity and impact on daily activities?

2. What is the one-year prognosis of knee complaints presented to the GP?

3. What are the factors predicting prognosis?

4. How are knee complaints managed by GPs?

The wide range of knee complaints included in our cohort study enables us to focus on specific aspects for specific subgroups and on the validity of measurement tools in a primary care setting. Therefore our secondary objectives for specific subgroups are as follows:

1. What is the predictive value of physical examination and history taking for detecting lesions that can be seen with Magnetic Resonance Imaging (MRI) in patients with acute traumatic knee injuries in General Practice?

2. What is the additive predictive value of MRI over physical examination and history taking for the prognosis of knee complaints in patients with acute traumatic knee injuries in General Practice?

3. What is the validity and responsiveness of disease specific questionnaire assessed disability measurements compared to device assessed disability measurements?

In this paper we will outline the composition of our cohort and define its subgroups. The objectives of our cohort demand that we give an accurate account of the population of patients that visit the GP with knee complaints. As we depended on active cooperation from the GPs for recruitment of patients, we need to ascertain that our cohort represents this population. Therefore objectives for the present paper are twofold:

1. To describe the methods used for data collection

2. To determine whether the recruitment procedures resulted in a patient selection that accurately represents the patients visiting the GP.

## Methods

### Design

This is a prospective, observational cohort study, with a follow-up period of one year. Data were collected using questionnaires and physical examinations. The researchers did not interfere with usual care with respect to advice, diagnostics or treatment. The study was approved by the ethics committee of the Erasmus Medical Centre Rotterdam.

### Inclusion criteria

Patients aged 12 years or above, consulting their GP for a new episode of knee complaints, were invited to participate in the study. New complaints were defined as complaints that were presented to the GP for the first time. Recurrent complaints for which the GP was not consulted within the last 3 months were also considered new complaints. Knee complaints that required urgent medical attention, such as fractures or infections were excluded. Patients with malignancies, neurological disorders or systemic musculoskeletal diseases that affect the outcome measures used in this study (i.e. Parkinson's disease, Rheumatoid Arthritis, Amyotrophic Lateral Sclerosis, etc.), as well as patients that were incapable of understanding the ramifications of participation, were excluded from participation.

### Recruitment

40 GPs from 5 municipalities in the southwest region of the Netherlands, connected to the Erasmus Medical Centre GP Research Network HONEUR and representing a total patient population of around 84.000 patients, participated in this prospective cohort study. We started recruitment in October 2001 in 1 municipality and a new municipality was added approximately every 3 months. All GPs recruited up to October 2003.

Patients were alerted to the existence of the study through posters in the waiting room. Participation of patients was voluntary and did not affect the care given to the patient. Patients received no compensation for participation. During consultation, the GP briefly informed the patient of the existence of the study and handed over written information and a baseline questionnaire. Interested patients forwarded their contact details to the researchers. The researchers contacted the patients to further inform patients of the study and to make an appointment for signing informed consent and performing a comprehensive standardized physical examination of both knees. Informed consent forms for minors (aged 12 through 17) were co-signed by a parent or guardian.

Participating GPs agreed to note the following items in their computerised medical files: relevant anamnestic findings, treatment details, a preliminary prognosis, and a diagnostic code from the International Classification of Primary Care (ICPC) [4], chosen from a list provided by the researchers.

### Physical examination

Two physiotherapists employed as research assistants (DC and EB) developed the standardized protocol for physical examination under supervision of two physiotherapists with over ten years of experience in both physiotherapy and research (SMAB and HW). Standardisation of the examinations among research assistants was accomplished by a series of training sessions before starting the inclusion of patients. These training sessions were repeated regularly over the course of the inclusion period. In total five physiotherapists (DC, EB, CV, AV and RvB) with clinical experience varying from one to 14 years performed the physical examinations of the patients.

The physical examination was planned as close to the date of consultation of the GP as possible. Irrespective of the type of symptoms presented, a standard range of tests was performed on both knees. The physical examination covered inspection of postural aspects, signs of inflammation, tests of swelling, locating tender areas, patellofemoral joint compression, crepitus, knee extensor and flexor strength, joint laxity, range of motion and meniscus tests (see table 1).

**Table 1:** Item list for physical examination

**inspection [18, 19]**	**palpation [18, 19]**	**specific diagnostic tests**
coloration	temperature	sustained flexion test [20]
valgisation/varisation	swelling: balottable patella sign	patellar grinding test [19]
overextension/limited extension	swelling: fluid shift/fluctuation sign	patellar axial pressure test [21]
tibial tuber swelling	pain tibial tuber	patellar apprehension test [21]
atrophy quadriceps	pain joint line	Steinmann II test [22]
flexion contracture hip	pain patellar edges	McMurray test [23]
internal/external rotation femur	pain patellar ligament	Apley's grind/traction tests [24]
internal/external rotation tibia	pain collateral lateral/medial ligaments	valgus/varus test [25]
foot pronation	pain insertion pes anserinus	anterior drawer test [25]
leg length difference	pain insertion iliotibial band	Lachman test [26]
	swelling fossa poplitea/Baker's cyst	pivot shift test [27]
**function assessment**	hypertrophy synovial plica	posterior drawer test [25]
flexion/extension active/passive [18, 19]	bursa prepatellaris pain/swelling	tibial posterior sag [28]
resisted flexion/extension [18, 19]	bursa infrapatellaris pain/swelling	

Discussion about diagnosis and/or appropriate management between patient and physiotherapist was discouraged, to avoid influencing the management initiated by the GP. The physical examination was repeated after one year, to enable comparison of perceived recovery with changes in test results.

### Self-report questionnaires

Baseline questionnaires were filled in by the patients before the baseline visit, and checked for completeness by the physiotherapist during the baseline visit after physical examination. Any uncertainties on behalf of the patient were discussed at that point and any necessary corrections made accordingly. The three monthly follow-up questionnaires were mailed to the participants, and returned by mail, except for the last questionnaire, which coincided with the follow-up physical examination. The questionnaires included possible prognostic factors as well as outcome measures. Details of questionnaire items are listed in table 2. For possible prognostic factors we enquired after socio-economic status, comorbidity, history of knee complaints, characteristics of the knee complaints, daily activities and coping behaviour. To determine whether the complaints were recurrent, we asked patients if they had experienced similar knee complaints in the past, with complaints disappearing at least several weeks before returning again now. We also asked if they had consulted the GP for that previous episode. Occupations were accredited with a level of knee loading ranging from 1 (e.g. office jobs) to 3 (e.g. construction workers and mail men) and sports activities with a level from 1 (strolling and swimming) to 5 (contact sports). Physical activities from level 2 upward are considered substantial knee loading sports activities. Contact sports and sports involving rapid changes of direction are considered heavy loading activities (levels 4 and 5).

**Table 2:** Questionnaire items

**item**	**evaluation at (months)**	**validation/reliability**
**demographics**		

age	0	-
gender	0	-
composition of the household	0	-
type of medical insurance	0	-
education level	0	-
comorbidity	0	-
**knee complaints**		
history, duration, recurrence, consultation previous episode, perceived cause of knee complaint, mechanism of traumatic injury	0	-
pain 11 point numeric rating scale	0, 3, 6, 9, 12	Likert scales have compared favourably to visual analogue scales for children and adults [5-7]
Lysholm knee scale [8]	0, 3, 6, 9, 12	developed for ligament ruptures, sensitive and reliable for meniscus tears, (patellar) chondral disorders [11, 12]
Knee Society Score [29]	0, 12	Intra/interobserver reliabililty poor [30]
- function score (patient questionaire)	0, 12	- function score moderate agreement
- knee score (observer, physical exam)	0, 12	- knee score poor agreement
WOMAC osteoarthritis index [9, 10]	0, 3, 6, 9, 12	validated and reliable for osteoarthritis [10]
pain and difficulty with cycling, running, jumping, squatting, kneeling	0, 3, 6, 9, 12	
**knee loading**		
daily activities: employment, volunteer jobs, household chores, study:	0, 3, 6, 9, 12	-
physical exercise/sports participation Frequency, intensity, duration, association with knee complaints	0, 12	-
**impact of knee complaint**		
hindrance during daily activities sick leave from daily activities	0, 3, 6, 9, 12	
**health related quality of life**		
SF-36 [31-33]	0, 3, 6, 9, 12	sensitive to change in common orthopaedic diagnoses [14], invalid for adolescents [15]
COOP/WONCA charts [34, 35]	0, 3, 6, 9, 12	valid for adults [34]
**treatment**		
advise given by the GP	0	-
knee medication, dose, frequency, duration, form of administration	0, 3, 6, 9, 12	-
medication for comorbidity	0, 3, 6, 9, 12	-
visits to health care professionals	follow-up	-
operations	follow-up	-
**Coping**		
Tampa Kinesiofobia Scale, (TKS) [36] catastrophizing	0	

Medical advice and interventions by the GP were recorded at baseline. During follow-up patients also recorded visits to other medical professionals with a short description of interventions.

### Outcome measures

Patients filled in their experienced recovery after one year on a 7 point Likert scale, ranging from 'fully recovered' to 'worse than ever'. Pain intensity was determined using a numeric rating scale (NRS) ranging from 0 (no pain) to 10 (unbearable pain). Numeric rating scales have compared favourably to visual analogue scales for children [5,6] as well as adults [7], though the number of points on these scales differed. Function assessments on disability level were determined using the Lysholm knee scoring scale (0–100) [8] and the WOMAC Hip and Knee Osteoarthritis Index (0–100) [9,10]. The Lysholm knee scoring scale was developed for ligament injuries, but was validated for use in various other knee disorders as well [11,12]. The questions from the WOMAC Hip and Knee Osteoarthritis Index were adapted to specifically address only the knee complaints.

The SF-36 was chosen for the assessment of health related quality of life because of its responsiveness [13] and its sensitivity to change in common orthopaedic diagnoses [14], though it has been shown to be invalid in adolescents [15]. We therefore also included the COOP/WONCA charts, which have not been validated in adolescents, but can be easily interpreted through illustrations. From the age of 18, patients filled in both SF-36 and COOP/WONCA charts, younger patients only filled in the COOP/WONCA charts.

### Definition of subgroups

As different pathologies are expected to show different prognoses, we defined three subgroups. Patients with non-traumatic knee complaints are divided into a group aged 12 to 35 (I) and a group aged 36 years and over (II), because around 35 years of age the predominance of specific diagnoses shifts from patellofemoral pain syndrome [16] to osteoarthritis [1]. The group of patients with traumatic knee complaints (III) includes all patients whose knee complaints were caused by a sudden impact or wrong movement within one year before consulting the GP. All other patients were considered to have non-traumatic complaints, based on the assumption that the immediate effects of traumatic injuries will have worn off after one year.

### Additional MRI

Patients aged between 18 and 65 years of age with an onset of trauma up to 5 weeks before consulting the GP were invited to participate in an additional MRI study. Participants were informed that patient and GP would not be informed of the presence or absence of detected lesions to prevent influencing the treatment strategy employed by the GP. Exceptions to this rule were lesions where urgent intervention was deemed necessary. For this additional study patients signed an additional informed consent form. MRI was performed between 3 to 6 weeks after the initial trauma, to allow swelling to subside while still observing the relatively acute stages of trauma. Following MRI a trained physiotherapist repeated the standardised physical examination. The assessors performing MRI and physical examination were blind to each other's results. The patients themselves recorded pain intensity and Lysholm score. After one year MRI and physical examination were repeated. If participants consulted medical specialists at a later date, the specialists were able to request MRI reports to prevent unnecessary duplication of diagnostic procedures.

### Device assessed disability

Adult patients with non-traumatic knee complaints living in three municipalities close to the research facility were invited to participate in the additional device assessed disability measurement using the Dynaport knee scoring system [17]. This system registers accelerations of torso, hip, upper and lower legs during simulations of daily activities like walking stairs, sitting down, or walking with grocery bags or loaded trolleys. Measurements were repeated after 6 months.

### Assessment of selective recruitment

To check whether the cohort adequately represents the patients that consulted the GP with a new episode of knee complaints during the inclusion period, the GPs computerized patient records were searched retrospectively for all occurrences of the relevant ICPC codes after the inclusion period had ended. As data collection from medical files is very labour-intensive, the search in each general practice was limited to a randomly assigned 4-month period within the total recruitment period. Within each municipality we made sure the 4-month periods covered all seasons. From all identified patients ICPC-code, diagnosis, age, sex and possible reasons for exclusion were registered anonymously on structured forms. ICPC-code and textual notes were both taken into account to determine whether onset of symptoms was considered traumatic by the GP. From the collected data we determined whether patients were eligible for inclusion in the cohort study. Eligible patients were then dichotomized according to age (12 to 35 years or above), gender, and traumatic onset of knee complaints. Participation rates within these dichotomized subgroups were compared using Odds Ratios (OR) and 95% confidence intervals, indicating their relative chances for inclusion into the study. Within each dichotomised subgroup we again compared the participation rates for the two other patient characteristics.

As the randomly chosen sample period was in itself a potential cause for bias of our analysis, we also compared the proportions of age, gender, and traumatic onset of knee complaints between participants from the sample periods and participants from the rest of the inclusion period. Furthermore, we compared proportions of ICPC codes in participants and non-participants from the sample periods, and in the participants of the sample periods and participants of the rest of the inclusion period, using Chi-square tests. For the statistical analyses we used SPSS for Windows, release 11.0.1.

## Study sample

### General cohort

Of the 1261 patients that forwarded their contact details to the researchers, 1068 (85%) signed informed consent. Reasons stated by contacted patients for non-participation are listed in table 3. The majority stated lack of time for participation (37%) or lack of personal gain (24%). The category miscellaneous included family circumstances, other health problems, language problems, and several patients judged themselves too old for participation. Ten patients were excluded: five because they were under 12 years of age, three because their complaints were not new, one because of rheumatoid arthritis and one patient was hospitalized with a bacterial infection of the knee.

**Table 3:** Reasons for non-participation of patients that forwarded their contact details to the researchers

	**N**
Lack of time/could get no time off from work/could not make an appointment for examination	72
No personal gain/too much bother	47
No longer any complaints at time of contact and no longer interested	19
Could not be contacted	15
Miscellaneous	30
Non-compliance with inclusion criteria	10

**Figure 1:**
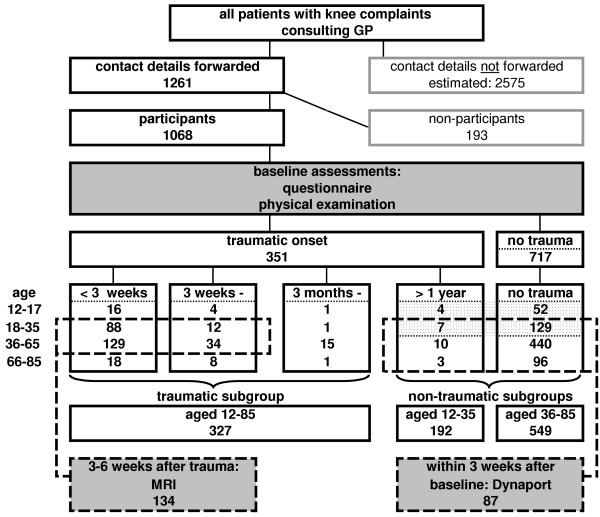
Patient recruitment and subgroup assignment.

**Figure 2:**
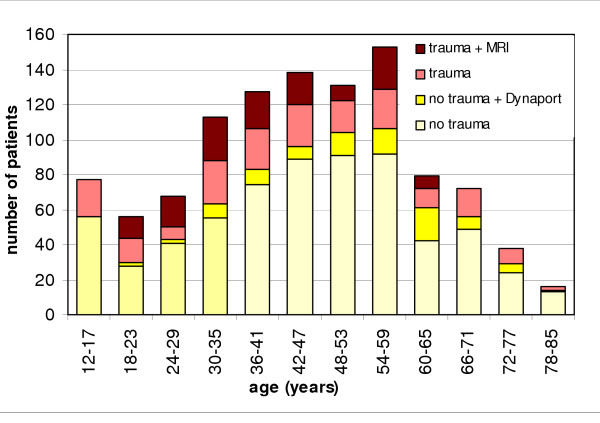
**Age distributions of subgroups. **Proportion of traumatic injuries and additional measurements per age category.

The flow diagram (fig. 1) shows the distribution of participants over different subgroups of the cohort and the additional measurements. 51% of the participants were assigned to the subgroup of non-traumatic knee complaints in patients aged over 35 years of age. 18% were assigned to the group of non-traumatic knee complaints in the age of 12 to 35 years. 31% were assigned to the traumatic knee injury subgroup. Figure 2 shows the age distribution of the entire cohort, identifying subgroups and additional measurements. The percentage of female participants in each subgroup was 50%, 47% and 44% respectively.

Of 1031 of the 1068 participants both questionnaire and physical examination results are available; 27 (2.5%) underwent a physical examination, but did not return their baseline questionnaire. Ten patients (0.9%) underwent no physical examination due to external circumstances like holidays and intervening commitments, but did return their baseline questionnaire. Data from the computerized medical files of 13 patients were not available.

### Additional assessments

Since starting inclusion for the MRI study in April 2002 there were 184 eligible patients, of which 134 patients participated. Reasons for non-participation were (in order of their contributions) unwillingness or inability to find time for these extra measurements, distance to the research facility, and the fact that detected lesions would only be reported to the patient and their GP if urgent intervention was deemed necessary.

Since starting inclusion for the knee function assessments study in August 2002 there were 330 eligible patients, of which 87 patients participated. Reasons for non-participation were unwillingness or inability to attend the extra visits required for these measurements.

### Patient selection

The search in the computerized patient records for occurrences of defined ICPC codes during the 4-month sample periods identified 310 eligible patients. 153 (49%) of those forwarded their contact details to the researchers, and 130 (42%) were included in the study and signed informed consent. The actual number of patients from which we received contact details during those same sample periods was 176, of which 150 patients were included in the cohort study (15% declined). When we looked up the medical files of the 150 participants we found that 20 of them lacked ICPC codes, explaining the 130 participants that were identified during the search. Likewise, a lack of ICPC coding in the medical records explains the discrepancy between the 176 contacted patients and the 153 that were identified in the search. Over the entire inclusion period the medical files of 15% of all participants lacked ICPC-codes.

**Figure 3:**
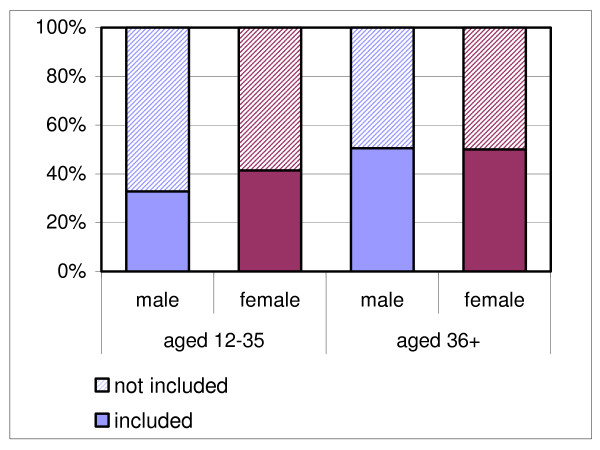
Inclusion rate of eligible patients per age group and gender.

**Figure 4:**
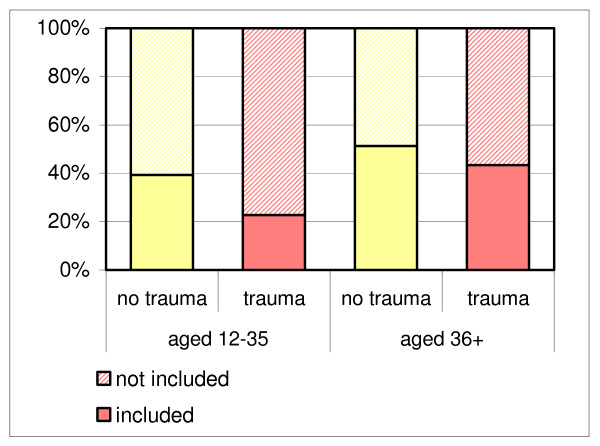
Inclusion rate of eligible patients per age group and traumatic onset.

Comparing the 130 participants and 180 non-participants identified through ICPC codes in the sample periods, we find significant selection with respect to age groups (table 4): we recruited relatively more patients over 35 years of age (OR 1.70; 1.15–2.77). This selective recruitment was more pronounced in the male population (OR 2.16; 1.12–4.18), than the female population (OR 1.22; 0.58–2.55). Overall, participation rates of women were not significantly higher than that of men (OR 1.13; 0.72–1.78). Participation rates of traumatic patients were lower than those of non-traumatic patients, though not significantly (OR 0.60; 0.26–1.43). Figures 3 and 4 show graphical representations of the proportions of included patients for each age group, subdivided for gender and traumatic onset of complaints.

**Table 4:** Patient characteristics of participants and non-participants

	**all participants in cohort**		**participants in sample**		**non-participants in sample**		**comparison^# ^of participation rates**
	**N**	**n** **(%)**	**N**	**n** **(%)**	**N**	**n** **(%)**	**OR** **(95% CI)**

**gender (n**_women_**)**	1045	494 (47%)	130	64 (49%)	180	83 (46%)	1.20 (0.78–1.85)
**age (n_>35 years_)**	1045	741 (71%)	130	94 (72%)	180	109 (61%)	1.79 (1.12–2.86)*
in men	551	380 (69%)	66	46 (69%)	97	50 (52%)	2.08 (1.11–3.93)*
in women	494	361 (73%)	64	48 (78%)	83	59 (71%)	1.41 (0.69–2.90)
in traumatic	197	134 (68%)	16	11 (73%)	34	17 (50%)	2.60 (0.80–8.98)
in non-traumatic	848	607 (72%)	114	83 (73%)	146	92 (63%)	1.63 (0.98–2.72)
**trauma^$^ (n_positive_)**	1045	197 (19%)	130	16 (12%)	180	34 (19%)	0.59 (0.32–1.09)
in men	551	109 (20%)	66	9 (14%)	97	20 (21%)	0.60 (0.26–1.38)
in women	494	88 (18%)	64	7 (11%)	83	14 (17%)	0.58 (0.23–1.47)
							
**ICPC codes**							
L15: unspecified		519 (51%)		69 (53%)		82 (46%)	
L78: acute distortion		107 (10%)		7 (5%)		20 (11%)	
L90: osteoarthritis		77 (7%)		9 (7%)		28 (16%)	
L94.2: Osgood-Schlatter		10 (1%)		1 (1%)		0 0%	
L96: acute meniscus/ligament ruptures		87 (8%)		12 (9%)		14 (8%)	
L97: chronic internal trauma		245 (23%)		32 (25%)		36 (20%)	

^$^ patient described onset of complaints in questionnaire as immediate, due to impact or twisting, maximally 1 year before consultation

^# ^comparing participation rates of age groups and traumatic injuries in sample

* p-value < 0.05

N total number of patients

n number of patients in a subset

When comparing participants from the sample periods with participants from the entire inclusion period, we found equal proportions of gender and age groups (see table 4). However, the proportions of traumatic injuries differed significantly: 12% of the patients in the sample periods were labelled 'traumatic injury' against 19% in the rest of the inclusion period (OR 0.59; 0.35 – 0.98).

We compared ICPC codes of participants and non-participants from the sample periods with a Chi-square test, pooling the codes L15 and L94.2 to prevent empty cells. We found a significant difference between the groups (Chi-statistic 11.2, p = 0.025). The differences are caused mainly by codes L78 and L96 for acute traumatic injuries and code L90 for osteoarthritis of the knee, all of which are less frequent in the participants. Comparison of ICPC codes of participants from the sample period with those of the rest of the inclusion period using the Chi-square test reveals no significant difference (Chi-statistic 5.6, p = 0.234).

## Discussion

We succeeded in starting a unique cohort study of patients with incident knee complaints in general practice. From October 2001 to October 2003 we included 1068 patients. Apart from its size, this cohort is unique in the range of knee complaints we studied: we included all ages from adolescents to the elderly, and we included both traumatic and non-traumatic complaints. Furthermore, this is the first cohort to include a standardised physical examination as well as questionnaires in patients who seek medical care for their knee complaints in general practice. We therefore think our cohort has a high potential for giving insight into the natural course of a range of knee complaints, and will give valuable information to base future effectiveness studies in primary care on. But in order to extrapolate the results of future publications ensuing from this cohort to clinical practice, we need to determine whether selective recruitment could induce bias.

### Selective recruitment

Patients below the age of 36 years were significantly less inclined to participate in our cohort study, and this trend was even stronger in the male population. Other comparisons did not produce statistically significant differences. However, the sample size may have been too small to prove that patients with traumatic injuries were underrepresented, again to a greater extent in the younger age group. Comparison of ICPC codes of the non-participants with those of the participants from the sample periods using a Chi-square test reveals a significant difference with respect to types of knee complaints. The difference is mainly caused by lower frequencies of the codes for the acute traumatic injuries L78 and L96, but lower frequencies of osteoarthritis of the knee (L90) also contribute.

The lack of ICPC codes in 15% of the participants indicates that our method for determining patient selection depends on the coding behaviour of the GPs. So is our sample a good representation of the situation during the entire inclusion period? We cannot identify the non-participants without ICPC codes to verify that, so we compared the proportions of gender, age groups and traumatic injuries of cohort and sample (table 4). We found a significantly smaller proportion of participants with traumatic injuries in the sample (12%) than in the cohort (19%) (OR 0.59; 0.35 – 0.98). As we made sure that the 4-month sample periods were distributed over all seasons in each municipality before randomly assigning them to the resident practices, we have ruled out seasonal fluctuations as a possible cause. But the working definition of 'traumatic injury' might explain something. In the medical records traumatic injuries can be recognised either by their ICPC code, or by the textual notes made by the GP. Some GPs tend to choose non-specific codes (L15) for any knee complaint, in which case recognition of traumatic injuries depends on the amount of detail in the textual notes. However, for further analyses in our cohort we use the patients perceived cause of the knee complaint together with the duration of the complaint to determine whether the complaint was of recent traumatic onset. With this definition we no longer detected any differences (29% in sample versus 31% in cohort). This indicates that the seemingly low participation rates of traumatic patients may have been an artefact caused by variations in the amount of detail in the medical files, rather than reflecting a non-representative sample. Furthermore, comparison of ICPC codes from the sample period with those of the rest of the inclusion period using the Chi-square test revealed no significant difference. One limitation remains: we have no insight into the possible differences in severity of knee complaints of participants and non-participants.

Comparing our results with those reported for the nationwide registration study [1], we found similar distributions of ICPC codes, suggesting that our population does not substantially deviate from patients with knee complaints in other Dutch general practices.

## Conclusion

Based on these results, we expect that the effects of selective recruitment will not cause significant bias, as future analyses will be performed separately for subgroups of patients, and adjustments will be made for gender and other possible risk factors and confounders.

We are confident that the present cohort study will provide new insights into the prognosis and management of knee complaints in primary care, and that the results can be extrapolated to all Dutch general practices.

## Competing interests

The author(s) declare that they have no competing interests.

## Authors' contributions

EMH coordinated the cohort study, data collection and data management, performed the statistical analyses and drafted the manuscript. SMAB conceived of the study, participated in its design and coordination, and helped to draft the manuscript. MYB recruited participating GPs. SMAB, MYB and BWK have helped in interpreting the data and critically revised the manuscript. All authors read and approved the final manuscript.

## Pre-publication history

The pre-publication history for this paper can be accessed here:


